# Analytical performance of the Xpert Carba-R assay for direct detection of carbapenemase genes in respiratory specimens from hospitalized patients: a multicenter evaluation in China

**DOI:** 10.3389/fmicb.2025.1709665

**Published:** 2025-12-10

**Authors:** Keke Li, Yuling Liu, Yihui Yao, Jie Wang, Yanxia Hui, Juan Li, Xiaoliang Luo, Xinghui Gao, Yi-Wei Tang, Lianhua Wei

**Affiliations:** 1 Department of Laboratory Medicine, Gansu Provincial Hospital, Lanzhou, China; 2 Department of Laboratory Medicine, The First Affiliated Hospital of Nanchang University, Nanchang, China; 3 School of Medicine, Zhongshan Hospital of Xiamen University, Xiamen, China; 4 Department of Laboratory Medicine, Hangzhou First People's Hospital, Hangzhou, China; 5 Department of Laboratory Medicine, Lanzhou University Second Hospital Dingxi Branch, Dingxi, China; 6 Department of Laboratory Medicine, Qingyang People's Hospital, Qingyang, China; 7 Department of Laboratory Medicine, Zhangye People's Hospital Affiliated to Hexi University, Zhangye, China; 8 Danaher Diagnostic Platform China/Cepheid, Shanghai, China; 9 College of Public Health, Chongqing Medical University, Chongqing, China

**Keywords:** carbapenem-resistant *Enterobacteriaceae*, genotypes, respiratory specimens, the Xpert Carba-R assay, whole-genome sequencing (WGS)

## Abstract

**Background:**

Respiratory tract infections remain the main sites of carbapenem-resistant *Enterobacteriaceae* (CRE) infections in China and limited information on enzyme genotypes and subtypes in China is available. In this study, we used whole-genome sequencing (WGS) and the Xpert Carba-R assay for the detection, genotyping and subsubtyping of CRE genes from respiratory specimens collected from Southeast (Zhejiang, Jiangxi and Fujian Provinces) and Northwest (Gansu Province) regions of China.

**Methods:**

Respiratory specimens from hospitalized patients clinically diagnosed with pneumonia from seven tertiary hospitals in China from October 2022 to November 2023 were included. The CRE enzyme genes in specimens were directly detected and genotyped by using the Carba-R assay. Carbapenem resistance and genotypes were compared to a referee in combination of phenotypical and genotypical analyses of pure cultures recovered from the respiratory specimen. The CRE enzyme subtypes in isolates were determined by the WGS.

**Results:**

A total of 433 respiratory specimens were collected and tested in this study. The Carba-R assay detected 180 *bla*KPC (41.3%), 64 blaNDM (14.8%), 5 blaIMP (1.2%), 4 blaVIM (0.9%), and 4 blaOXA-48 (0.9%) genes. In comparison to the above referee, the sensitivity and specificity of the Carba-R assay for CRE detection were 100 and 90.4%. While there were no genotype distribution differences between the Southeast and Northwest regions, more *KPC* subtypes were identified in the Northwest (*n* = 5) than Southeast (*n* = 2) region.

**Conclusion:**

The Carba-R assay satisfactorily detected and identified carbapenemase genes directly in the respiratory specimens. A variety of CRE enzyme genotypes and subtypes were identified in respiratory specimens especially the Northwest region of China.

## 1 Introduction

The dissemination of carbapenem-resistant *Enterobacteriaceae* (CRE) represents a major global public health threat. In 2015, the U.S. Centers for Disease Control and Prevention (CDC) defined CRE as *Enterobacteriaceae* that exhibit *in vitro* resistance to at least one carbapenem (Centers for Disease Control and Prevention, n.d.). According to data from the World Health Organization (WHO) Global Antimicrobial Resistance Surveillance System (GLASS) in 2021, the global detection rate of CRE remains high, with resistance rates to meropenem and imipenem among carbapenem-resistant *Klebsiella pneumoniae* (CRKP) isolates reported at 12.34 and 10.63%, respectively (World Health Organization, 2021). Data from the China Antimicrobial Surveillance Network (CHINET, http://www.chinets.com) indicate that the resistance rates of *Enterobacteriaceae* to meropenem and imipenem increased from 2.1 and 3.1% in 2005 to 12.5 and 12.3% in 2023, respectively. Meanwhile, the prevalence of CRKP increased markedly from 2.9% in 2005 (imipenem resistance) to 25.0% in 2018, followed by a slight decline to 22.6% in 2022 (Qin et al., 2024).

High-risk populations for CRE infection typically include patients admitted to intensive care units, as well as those hospitalized in hematology, oncology, infectious diseases, transplantation, neurology, and surgical wards (Moghnieh et al., 2021; Saidel-Odes et al., 2024). In China, pulmonary infections account for the majority of CRE infections, with more than 65% of patients presenting with lower respiratory tract involvement, followed by urinary tract, intra-abdominal, and bloodstream infections. However, the prevalence varies substantially across different regions (Zhang et al., 2018). The high global mortality associated with CRE infections underscores the urgency of ongoing epidemiological surveillance. Respiratory infections caused by CRE are of particular concern among patients with hospital-acquired pneumonia (HAP), especially ventilator-associated pneumonia (VAP), which frequently serve as epicenters of nosocomial outbreaks. Delayed or inappropriate antimicrobial therapy during the early stages of infection can result in poor clinical outcomes and increased mortality (Chen et al., 2021; Tuon et al., 2017). A large multicenter cohort study demonstrated that infections caused by CRKP strains producing KPC-2 or KPC-3 enzymes are associated with mortality rates approaching 40% among patients with lower respiratory tract infections (Tumbarello et al., 2015). Similarly, a large-scale database study conducted in the United States reported that undiagnosed community-onset CRE infections were associated with a fourfold increase in the risk of inappropriate therapy, leading to higher mortality, prolonged hospital stays, and increased healthcare costs (Zilberberg et al., 2017). In the absence of bacterial whole-genome sequencing, Carbapenemase typing remains one of the essential prerequisites for guiding appropriate antimicrobial therapy. Novel carbapenem–*β*-lactamase inhibitor combinations, such as Ceftazidime–avibactam (CZA) (Sanz Herrero, 2022), Meropenem–vaborbactam, and Imipenem–relebactam (Zhanel et al., 2018), have been developed to overcome carbapenemase-mediated resistance. However, these agents exhibit activity primarily against class A and D serine *β*-lactamases, while remaining ineffective against class B metallo-β-lactamases (MBLs).

Until recently, several methods have been available for detecting carbapenemase production, including the modified carbapenem inactivation method (mCIM), EDTA-carbapenem Inactivation Method (eCIM), immunochromatographic assay NG-Test CARBA 5 (CARBA 5), Color developing immunoassay test (CDI) (Zhang et al., 2022), Rapidec Carba NP, and polymerase chain reaction (PCR)-based assays (Shaikh et al., 2020). However, these approaches generally require prior isolation and purification of bacterial colonies from clinical specimens, followed by antimicrobial susceptibility testing and subsequent detection of carbapenemase genes in confirmed CRE isolates. This multi-step workflow typically takes 24–72 h, which significantly limits its clinical utility for guiding timely antimicrobial therapy in patients with active infections or CRE colonization. The Xpert Carba-R assay provides a promising alternative by enabling highly sensitive and specific detection of carbapenemase-producing *Enterobacteriaceae* directly from clinical specimens (Cury et al., 2020). It has been successfully validated for diverse polymicrobial samples, including abdominal drainage fluid and blood (Cortegiani et al., 2016; Cointe et al., 2019). Nevertheless, robust clinical evidence supporting its rapid and reliable use for the direct detection of CRE in respiratory specimens remains limited.

The Xpert Carba-R assay (Cepheid, Sunnyvale, CA, USA) enables rapid and reliable detection of the five major carbapenemase genes-*bla*_KPC_, *bla*_NDM_, *bla*_VIM_, *bla*_IMP_, and *bla*_OXA-48_-within approximately 1 h serving as a qualitative molecular diagnostic tool for carbapenemase-producing *Enterobacteriaceae*. In China, respiratory tract infections constitute the predominant clinical presentation of CRE; however, data regarding the regional distribution and molecular subtypes of carbapenemase genes remain limited (Ma et al., 2023). In this study, we combined WGSwith the Xpert Carba-R assay to characterize the genotypes and subtypes of carbapenemase genes directly from respiratory specimens collected across southeastern (Zhejiang, Jiangxi, and Fujian Provinces) and northwestern (Gansu Province) China. This work provides valuable insights into the molecular epidemiology of carbapenemase gene dissemination and offers potential diagnostic strategies for the rapid detection and molecular surveillance of CRE-associated respiratory infections.

## 2 Materials and methods

### 2.1 Specimen collection and preparation

**Figure 1:**
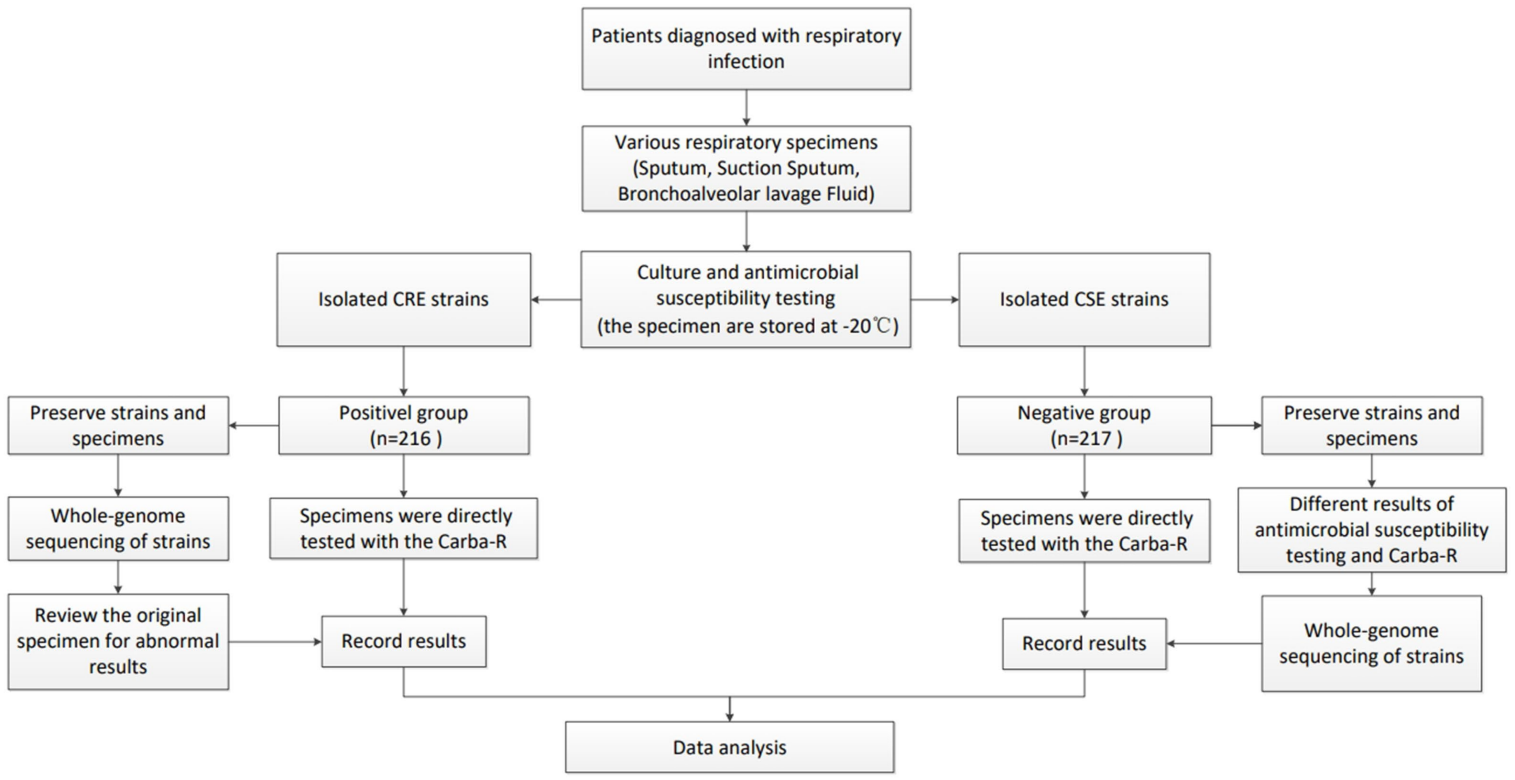
Flow chart of the experimental processes.

This study enrolled patients with clinically diagnosed pneumonia from seven tertiary care hospitals in China between October 2022 and November 2023. Respiratory specimens included bronchoalveolar lavage fluid, aspirated sputum, and expectorated sputum. For expectorated sputum, Gram staining was performed prior to inoculation to assess sample quality and exclude inadequate specimens. After inoculation, all samples were aliquoted and stored at −80 °C: one aliquot underwent the Carba-R assay testing following antimicrobial susceptibility testing, while the other was preserved for long-term storage and subsequent validation. Clinical and microbiological data were recorded for all enrolled specimens, including patient demographics (sex and age), hospital department, admission number, bacterial species, and antimicrobial susceptibility profiles. The flowchart of data collection is shown in Figure 1.

### 2.2 Bacterial culture and isolate identification

Respiratory specimens were cultured on Columbia agar supplemented with 5% sheep blood (Antu, Zhengzhou, China) and MacConkey agar (Oxoid, Hampshire, UK) for pathogen detection and isolation. After incubation at 35 °C for 24 h in 5% CO₂, presumptive colonies were selected and identified by matrix-assisted laser desorption ionization–time of flight mass spectrometry (MALDI-TOF MS; Bruker Daltonics, Bremen, Germany, or bioMérieux, Marcy l’Etoile, France). Antimicrobial susceptibility testing was performed using the Vitek 2 system with AST-GN334 cards (bioMérieux). Quality control of susceptibility testing was ensured with *Escherichia coli* ATCC 25922, *Klebsiella pneumoniae* ATCC BAA-1705, and *K. pneumoniae* ATCC BAA-1706. Screening for CRE and interpretation of susceptibility results followed the Clinical and Laboratory Standards Institute (Clinical and Laboratory Standards Institute. 2023. *M100: performance standards for antimicrobial susceptibility testing.* 33st Edition. Clinical and Laboratory Standards Institute, Wayne, PA, USA) guidelines. Carbapenem resistance was defined as any minimum inhibitory concentration (MIC) at or above the CLSI resistance breakpoint for *Enterobacterales*. All participating hospitals had successfully completed the external quality assessment program organized by the National Center for Clinical Laboratories (NCCL), which conducts high-level proficiency testing for bacterial identification and antimicrobial susceptibility three times annually.

### 2.3 WGS and analysis

All CRE isolates were submitted to the JINSHI Medical Testing Center (Tianjin, China) for genomic DNA extraction and WGS. WGS was performed using Illumina (Illumina, San Diego, CA, USA) short-read sequencing technology, and genome assembly was conducted with SPAdes software (version 3.12.0) using the complete set of sequencing reads. Annotation of antibiotic resistance genes, virulence factors, plasmid replicons, serotype predictions, and multilocus sequence types (MLST) was achieved by sequence alignment against the comprehensive antibiotic resistance database (CARD), and NCBI nucleotide (NT) databases using BLAST (version 2.9.0+).

### 2.4 The Xpert Carba-R assay

The GeneXpert instrument system (Cepheid, Sunnyvale, CA, USA) is a fully automated real-time quantitative PCR (qPCR) platform. One of its functions is the detection of five carbapenemase genes *bla*_KPC_, *bla*_NDM_, *bla*_VIM_, *bla*_OXA-48_, and *bla*_IMP_. Before using the Carba-R assay to enter the Xpert cartridge, all clinical respiratory specimens need to be preprocessed. The specimen preprocessing process can refer to the previously described (Cai et al., 2020), but we have optimized the sample addition process. During the liquefaction process of clinical respiratory specimens, MTB sample processing solution can be used for specimen digestion. Add 2 mL of the sample to the Xpert MTB/RIF sample processing solution and mix for more than 15 min. After multiple shaking cycles until the sample is completely liquefied, transferred into 1 mL of the mixture to the Carba-R assay sample reagent, and then transfer 1.7 mL of the mixture to the Carba-R assay for testing. The Carba-R assay was performed on all validation specimens according to the manufacturer’s package insert, and results were interpreted directly from the report generated by the GeneXpert instrument. Internal Probe Inspection Control (PCC) and Sample Processing Control (SPC) serve as quality controls for the Xpert analysis process, ensuring the accuracy of reagent and sample processing.

### 2.5 Ethics statement

This study does not include factors that require patient consent, therefore all participating hospitals waived the need for a written informed consent from included patients. In addition, the design and reporting of this study have been approved by the local institutional review committee. This is a pure laboratory intervention without patient involvement. Samples employed in the analyses were de-identified before access.

### 2.6 Data analysis and statistics analysis

According to the antimicrobial susceptibility testing results, specimens yielding CRE isolates were classified as the positive group, whereas those yielding carbapenem-susceptible *Enterobacteriaceae* (CSE) isolates were classified as the negative group. The inclusion requirement for the negative group is that the sample with the closest detection time to the positive group, and the isolated CSE strain type must be consistent with the positive group. The Carba-R assay of positive result means that at least one *bla*_KPC_, *bla*_NDM_, *bla*_VIM_, *bla*_OXA-48_, or *bla*_IMP_ carbapenemase gene was detected in this specimen. A negative result means that there were no carbapenemase genes detected by the Carba-R assay. Each specimen included in the study was calculated the Test turnaround time (TAT).

In the positive group, WGS results of the isolated CRE strains were employed as the reference standard, whereas in the negative group, antimicrobial susceptibility testing (AST) results of the isolates served as the reference benchmark. According to these criteria, the sensitivity, specificity, and other pertinent statistical parameters of the Xpert Carba-R assay for the direct detection of carbapenemase genes in respiratory specimens were evaluated. All statistical analyses were performed using the Statistical Package for the Social Sciences (SPSS) version 19.0.

## 3 Results

Between October 2022 and November 2023, a total of 433 respiratory specimens from patients diagnosed with pneumonia, with 216 specimens in the positive group and 217 specimens in the negative group. The specimens were collected from Lanzhou, Dingxi, Qingyang, and Zhangye in Gansu Province of Northwest regions in china, as well as Hangzhou in Zhejiang Province, Nanchang in Jiangxi Province, and Xiamen in Fujian Province of Southeast regions in china. Among all specimens, sputum and aspirated sputum accounted for 74.8%, while bronchoalveolar lavage fluid accounted for 25.2% males accounted for 258 (59.6%) and females also constituted 175 (40.4%), with an age range from 16 to 92 years.

**Figure 2:**
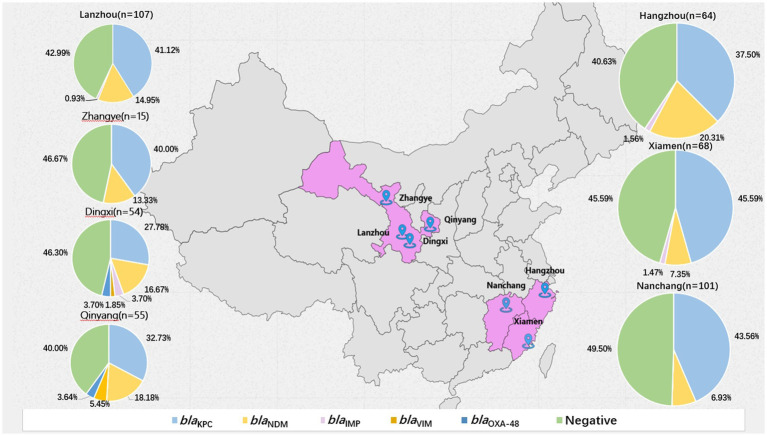
Distribution of carbapenemase genes in seven locations in China determined by Carba R.

Among all the respiratory specimens included in the study, 216 specimens cultured CRE strains, with each specimen cultivating only one CRE strain, 217 specimens did not culture CRE strains. Negative specimens were selected to match the bacterial strain and department of the positive specimens, with a time difference of no more than 3 days. Direct the Carba-R assay testing was performed on all 433 respiratory specimens included in the study. In the positive group the direct results of the Carba-R assay detection were 152 *bla*_KPC_,33 *bla*_NDM_, 1 *bla*_IMP_, 1 *bla*_VIM_, 20 *bla*_KPC_-*bla*_NDM_,2 *bla*_KPC_-*bla*_IMP_, 2 *bla*_NDM_-*bla*_IMP_, 2 *bla*_KPC_-*bla*_OXA-48_,1 *bla*_KPC_-*bla*_NDM_-*bla*_OXA-48_,1 *bla*_VIM_-*bla*_NDM_-*bla*_OXA-48_ and 1 negative. In the negative group, the Carba-R assay detected 2 *bla*_KPC_, 6 *bla*_NDM_, 2 *bla*_VIM_, and 1 *bla*_KPC_-*bla*_NDM_, while the remaining206 specimens did not detect any carbapenemase genes. The single-gene classification results were 180 *bla*_KPC_ (41.6%), 64 *bla*_NDM_ (14.8%), 5 *bla*_IMP_ (1.2%), 4 *bla*_VIM_ (0.9%), and 4 *bla*_OXA-48_ (0.9%) genes. In all regions, the detection rate of *bla*_KPC_ was the highest, ranging from a minimum of 28.8% to a maximum of 45.6%, followed by *bla*_NDM_, with a detection rate ranging from 6.9 to 20.3%. Detailed results are shown in Figure 2.

By comparing with the referee method, the Carba-R assay direct detection method for carbapenemase genes in specimens demonstrated high sensitivity and specificity. The sensitivity and specificity for *bla*_KPC_ were 99.4 and 95.8%, for *bla*_NDM_ were 97.2 and 92.7%, and the specificity for *bla*_VIM_, *bla*_IMP,_ and *bla*_OXA-48_ was 99.1%. Regardless of the carbapenemase gene, the Carba-R assay achieved 100% sensitivity and 90.4% specificity for direct detection respiratory specimens, as shown in Table 1. While the Carba-R assay method detected a small number of positive samples for *bla*_IMP_, *bla*_VIM_, and *bla*_OXA-48_, the referee method found only one sample containing the *bla*_IMP_ gene, with no detection of *bla*_IMP_, *bla*_VIM_, or *bla*_OXA-48_ genes in the other samples.

**Table 1:** Sensitivity and specificity of Xpert Carba-R detection for carbapenem resistance genes (*n* = 433).

Carbapenemase gene	No. of specimens by result				Sensitivity (%)	Specificity (%)
	R+ X+	R+ X−	R− X+	R− X−		
*bla*_KPC_	169	1	11	252	99.4	95.8
*bla*_NDM_	35	1	29	368	97.2	92.7
*bla*_VIM_	0	0	4	429	NA	99.1
*bla*_IMP_	1	0	4	428	NA	99.1
*bla*_OXA-48_	0	0	4	429	NA	99.1
Any gene	204	0	22	207	100	90.4

R, referee; X, Xpert Carba-R assay; +, detected; −, not detected; NA, not applicable.

In this included 219 specimens from Southeast China (Hangzhou, Nanchang, Xiamen) and 214 specimens from Northwest China (Gansu), with only a 5-specimen difference between regions. The detection of the *bla*_KPC_ gene in t the Carba-R assay in Southeast regions (*n* = 98) was higher than in the Northwest region (*n* = 84), while the detection of the *bla*_NDM_ gene (*n* = 28) was less frequent in Northwest region (*n* = 36). Performance metrics showed sensitivity and specificity for *bla*_KPC_ in the Southeast and Northwest regions were 100, 98.7 and 97.6%, 94.2% respectively, with sensitivity and specificity for *bla*_NDM_ were 100, 96 and 91.8%, 93.7%. Comparative analysis between WGS and the Carba-R assay revealed discrepancies in carbapenemase detection among clinical isolates from Northwestern China. WGS identified NDM-7and KPC-66 genes in one specimen isolate, while the Carba-R assay only detected NDM in specimen. Additionally, WGS confirmed NDM-1 in specimen isolate were reported as KPC positive by the Carba-R assay in same specimen. Chi-square testing showed no statistically significant differences in the Carba-R assay’s sensitivity and specificity between the two regions (*χ*^2^ test, *p* > 0.05), indicating consistent assay performance despite these genotypic-phenotypic discordances (Table 2).

**Table 2:** Sensitivity and specificity of Carba-R assay between Gansu and Zhejiang/Fujian/Jiangxi.

Strain type (*n* = 433)	Genotype	No. of specimens by result				Sensitivity (%)	*X^2^ p-*value (<0.05)	Specificity (%)	*X^2^ p-*value (<0.05)
		R+ X+	R+ X−	R− X+	R− X−				
Southeast	*bla*_KPC_	95	0	3	121	100	*bla*_KPC_	97.6	*bla*_KPC_
(ZJ/FJ/JX *n* = 219)	*bla*_NDM_	11	0	17	191	100	0.454	91.8	0.345
Northwest	*bla*_KPC_	74	1^a^	8	131	98.7	*bla*_NDM_	94.2	*bla*_NDM_
(Gansu *n* = 214)	*bla*_NDM_	24	1^b^	12	177	96	0.422	93.7	0.539

a kpc-66 as identified by WGS.

b NDM-1 as identified by WGS.

WGS was performed on a total of 227 isolates, including all CRE strains isolated from the positive group and the Carba-R assay positive specimens from the negative group. The WGS results for the negative group were uniformly negative. In the positive group, the following carbapenemase gene variants were identified: 163 KPC-2, 3 KPC-9, 1 KPC-18, 1 KPC-19, 1 KPC-55, 1 KPC-66, 23 NDM-1, 10 NDM-5, 1 NDM-6, 1 NDM-7, 1 NDM-9, and 1 IMP-79 and 1 negetive (Table 3). Regional analysis revealed KPC-2 as the predominant genotype, with higher detection rates in Southeast China but greater KPC subtype diversity in the Northwest. Among NDM variants, NDM-1 predominated and showed elevated prevalence in Northwest China. The WGS-detected carbapenemase gene subtypes demonstrated distinct geographic distributions across all seven study sites.

**Table 3:** Genotype and subtype distributions between Northwest and Southeast regions.

Carbapenemase genes identified on WGS		No. of isolates		
Genotype	Subtypes	*Gansu*	ZJ/FJ/JX	Total
*bla*_KPC_	*bla*_kpc-2_	70	93	163
	*bla*_kpc-9_	1	2	3
	*bla*_kpc-18_	1	0	1
	*bla*_kpc-19_	1	0	1
	*bla*_kpc-55_	1	0	1
	*bla*_kpc-66_	1	0	1
*bla*_NDM_	*bla*_NDM-1_	15	8	23
	*bla*_NDM-5_	8	2	10
	*bla*_NDM-6_	0	1	1
	*bla*_NDM-7_	0	1	1
	*bla*_NDM-9_	1	0	1
*bla*_IMP_	*bla*_IMP-79_	1	0	1

## 4 Discussion

The convenience, rapidity, and simplicity of the Xpert Carba-R assay make it a promising tool for the direct detection and identification of CRE in lower respiratory tract specimens. In this study, we evaluated the diagnostic performance of the Carba-R assay for detecting carbapenemase genes directly from clinical samples and further analyzed its utility in respiratory specimens collected from pneumonia patients in two distinct geographic regions of China, namely the Southeast (Zhejiang, Jiangxi, and Fujian) and the Northwest (Gansu). Although no regional differences in the distribution of carbapenemase gene subtypes were observed between the two regions, the molecular epidemiological characterization of CRE isolates provided new epidemiological data to support the prevention and control of CRE in China.

Previous studies have demonstrated that the Carba-R assay exhibits excellent positive percent agreement, negative percent agreement, and concordance rates for the direct detection of carbapenemase genes from clinical specimens, particularly sputum samples (Cai et al., 2020). In our study, the sensitivity and specificity of the Carba-R assay positive specimens for *bla*_KPC_ were 99.4 and 95.8%, respectively, while those for *bla*_NDM_ were 97.2 and 92.7%. Although the sensitivity and specificity for *bla*_NDM_ were slightly lower in comparison, they still hold significant clinical value. For *bla*_VIM_, *bla*_IMP_, and *bla*_OXA-48_, sensitivity could not be calculated due to the small number of detected cases; however, all exhibited high specificity. Therefore, the Carba-R assay can serve as an effective negative screening tool for these three genotypes when applied directly to respiratory specimens. Among the positive cases, specimens detected with multiple carbapenemase genotypes by the Carba-R assay often yielded isolates carrying only a single genotype, which affected the specificity results, particularly for *bla*_NDM_. Although WGS was not performed for all isolates recovered from the negative group, we conducted WGS analysis on discrepant isolates identified by the Carba-R assay to ensure the reliability of our findings.

In this study, for specimens showing multiple carbapenemase genotypes as well as a small number of carbapenemase genes detected in the negative group, we considered the possibility of missed detection by conventional bacterial culture. This may occur when non-resistant colonies are selected for antimicrobial susceptibility testing, or when bacteria harbor resistance genes that are either not expressed or expressed only at low levels (Qin et al., 2024; Stasiak et al., 2021). Other possibilities include prior antibiotic treatment in patients or the detection of carbapenemase genes originating from non-*Enterobacteriaceae* organisms. The likelihood of co-infections involving multiple carbapenemase-producing strains is also common in respiratory tract infections, particularly among long-term hospitalized patients; for example, co-infections with CRKP and carbapenem resistant *Acinetobacter baumannii* (CRAB) or carbapenem resistant *Pseudomonas aeruginosa* (CRPA) have been reported (Li et al., 2021), which may explain the detection of multiple resistance genes in some specimens. In our study, a few cases involving *bla*_IMP,_ *bla*_VIM_, and *bla*_OXA-48_ genotypes were identified. Previous studies have shown that *bla*_IMP_ can be detected in *K. pneumoniae, E. cloacae, K. oxytoca*, and *P. rettgeri* strains in China, while OXA-48 is rarely reported and mainly restricted to *K. pneumoniae* (Han et al., 2020; Zhang et al., 2018). However, sequencing of the corresponding isolates did not confirm the presence of these genes, which may also be attributable to the aforementioned reasons.

Carbapenem resistance mediated by carbapenemase production represents the most prevalent mechanism among CRE worldwide. In this study, specimens from seven hospitals across different geographic regions of China were included, reflecting varying CRE isolation rates. Among the 433 isolates, the following species were identified: *K. pneumoniae* (*n* = 356), *E. coli* (*n* = 18), *E. cloacae* (*n* = 32), *K. oxytoca* (*n* = 6), *P. mirabilis* (*n* = 4), *S. marcescens* (*n* = 6), *E. aerogenes* (*n* = 3), *C. freundii* (*n* = 2), and *P. rettgeri* (*n* = 4). A total of 215 isolates were classified as CRE. WGS analysis of 170 CRKP isolates revealed that 90.9% carried *bla*_KPC-2_ (*n* = 160), 1.9% carried *bla*_KPC-9_ (*n* = 3), and 2.8% carried *bla*_NDM-1_ (*n* = 5). The prevalence of *bla*_KPC-2_ in our study was markedly higher than that reported in previous multicenter studies in China (Shi et al., 2024; Chen et al., 2024), which may be related to the nationwide increase in CRE detection—particularly CRKP—following the complete lifting of COVID-19 restrictions in December 2022 (Liu et al., 2024; Dabaja-Younis et al., 2024). Among the remaining Enterobacterales isolates (*n* = 39), the predominant carbapenemase genotypes were NDM variants, with *bla*_NDM-1_ (46.2%, *n* = 18) and *bla*_NDM-5_ (25.6%, *n* = 10) being most common. Although more KPC and NDM subtypes were identified in the Northwest compared to the Southeast region, the sensitivity and specificity of the Carba-R assay for detecting *bla*_KPC_ and *bla*_NDM_ directly from respiratory specimens did not differ significantly between regions. This finding highlights the potential for widespread application of the assay across different regions of China. Importantly, our results demonstrate that the Carba-R assay can simultaneously detect five classes of carbapenemase genes, covering multiple subtypes, within 2–4 h (Liao et al., 2021). With its high degree of automation and suitability for near-patient testing, this method provides a practical and effective tool for respiratory CRE screening in clinical settings.

CRE respiratory tract infections are a major cause of mortality and are associated with high fatality rates (de Maio Carrilho et al., 2016). Invasive procedures, such as mechanical ventilation, often serve as potential portals of entry for CRE into the lungs of pneumonia patients, thereby providing a plausible explanation for the subsequent development of CRE infections (Gao et al., 2019). Consequently, early screening for CRE colonization in such patients is of great clinical importance, as these represent modifiable risk factors that can be addressed through infection prevention and control strategies.

Previous studies have reported that the clinical outcomes of patients with extraintestinal CRE colonization are comparable to those of patients with confirmed CRE infections at the same anatomical site. Among these, respiratory tract colonization has been associated with the poorest outcomes and the highest 30-day mortality rates (Howard-Anderson et al., 2022). Similarly, another study demonstrated that CRE respiratory colonization and CRE infection yielded comparable overall clinical outcomes, 30-day mortality, and 90-day readmission rates, all of which necessitate routine clinical management (van Duin et al., 2020). These findings support our recommendation that CRE colonization in the respiratory tract should also warrant therapeutic intervention.

In addition, emerging evidence suggests that the cycle threshold (CT) values obtained directly from the Carba-R assay testing of bronchial aspirates may help distinguish CRE infection from colonization (Burillo et al., 2016), although further validation studies are still required. Moreover, compared with culture-based methods, the Carba-R assay has demonstrated substantial cost-effectiveness in the early identification of colonization or infection (Toumi et al., 2025), although these data are limited to rectal swab specimens. To date, no studies have specifically evaluated the use of the Carba-R assay for early screening of carbapenemase-producing Enterobacterales (CPE) in respiratory samples. Our present study, which included only CRE isolates recovered from respiratory specimens, therefore provides preliminary evidence that may serve as a theoretical basis for future validation of the Carba-R assay as a novel diagnostic approach.

Future studies will focus on validating the utility of this assay for early intervention in patients with CRE respiratory tract colonization or infection. Key outcomes to be evaluated include its impact on timely clinical recognition and infection markers, optimization of antimicrobial therapy, and reductions in treatment duration and antibiotic use. We will also assess patient-centered outcomes such as duration of mechanical ventilation, ICU and hospital stay, *C. difficile*-associated diarrhea, and mortality, as well as cost-effectiveness with respect to antibiotic expenditures. These investigations will clarify the role of the Carba-R assay as both a rapid diagnostic tool and a means to support antimicrobial stewardship.

## 5 Conclusion

In summary, our study confirms that the Xpert Carba-R assay is a highly sensitive and reasonably specific method for rapid detection of carbapenemase-producing organisms directly from respiratory specimens. It is implementation could facilitate timely infection control interventions and guide empirical therapy in clinical microbiology laboratories. Nevertheless, the assay’s limitations in detecting rare variants and mixed genotypes underscore the complementary role of WGS, especially in epidemiological investigations and outbreak surveillance.

## Data Availability

The original contributions presented in the study are included in the article/supplementary material, further inquiries can be directed to the corresponding authors.
